# Corticotroph Aggressive Pituitary Tumors and Carcinomas Frequently Harbor ATRX Mutations

**DOI:** 10.1210/clinem/dgaa749

**Published:** 2020-10-27

**Authors:** Olivera Casar-Borota, Henning Bünsow Boldt, Britt Edén Engström, Marianne Skovsager Andersen, Bertrand Baussart, Daniel Bengtsson, Katarina Berinder, Bertil Ekman, Ulla Feldt-Rasmussen, Charlotte Höybye, Jens Otto L Jørgensen, Anders Jensen Kolnes, Márta Korbonits, Åse Krogh Rasmussen, John R Lindsay, Paul Benjamin Loughrey, Dominique Maiter, Emilija Manojlovic-Gacic, Jens Pahnke, Pietro Luigi Poliani, Vera Popovic, Oskar Ragnarsson, Camilla Schalin-Jäntti, David Scheie, Miklós Tóth, Chiara Villa, Martin Wirenfeldt, Jacek Kunicki, Pia Burman

**Affiliations:** 1 Department of Immunology, Genetics and Pathology, Uppsala University, Uppsala, Sweden; 2 Department of Clinical Pathology, Uppsala University Hospital, Uppsala, Sweden; 3 Department of Pathology, Odense University Hospital, Odense, Denmark; 4 Department of Clinical Research, University of Southern Denmark, Odense, Denmark; 5 Department of Medical Sciences, Endocrinology and Mineral Metabolism, Uppsala University, Uppsala, Sweden; 6 Department of Endocrinology and Diabetology, Uppsala University Hospital, Uppsala, Sweden; 7 Department of Endocrinology, Odense University Hospital, Odense, Denmark; 8 Clinical Institute, University of Southern Denmark, Odense, Denmark; 9 Department of Neurosurgery, Foch Hospital, Suresnes, France; 10 Department of Internal Medicine, Kalmar, Region of Kalmar County, Sweden; 11 Department of Biomedical and Clinical Sciences, Linköping University, Linköping, Sweden; 12 Department of Molecular Medicine and Surgery, Karolinska Institute, Stockholm, Sweden; 13 Department of Endocrinology, Karolinska University Hospital, Stockholm, Sweden; 14 Department of Endocrinology, University Hospital, Linköping, Sweden; 15 Department of Health, Medicine and Caring Sciences, Linköping University, Linköping, Sweden; 16 Department of Medical Endocrinology and Metabolism, Rigshospitalet, Copenhagen, Denmark; 17 Institute of Clinical Medicine, Faculty of Health Research Sciences, Copenhagen University, Copenhagen, Denmark; 18 Department of Endocrinology and Internal Medicine, Aarhus University Hospital, Aarhus, Denmark; 19 Section of Specialized Endocrinology, Department of Endocrinology, Oslo University Hospital, Oslo, Norway; 20 Faculty of Medicine, University of Oslo, Oslo, Norway; 21 Centre for Endocrinology, William Harvey Research Institute, Barts, UK; 22 The London School of Medicine and Dentistry, Queen Mary University of London, London, UK; 23 Department of Endocrinology and Metabolism, Copenhagen University Hospital, Copenhagen, Denmark; 24 Mater Infirmorum Hospital, Belfast Health & Social Care Trust (BHSCT), UK; 25 Regional Centre for Endocrinology and Diabetes, Royal Victoria Hospital, Belfast Health & Social Care Trust, UK; 26 Patrick G Johnston Centre for Cancer Research, Queen’s University, Belfast, UK; 27 Department of Endocrinology and Nutrition, UCL Cliniques universitaires Saint-Luc, 1200 Brussels, Belgium; 28 Institute of Pathology, School of Medicine, University of Belgrade, Belgrade, Serbia; 29 University of Oslo (UiO) and Oslo University Hospital (OUS), Department of Pathology, Translational Neurodegeneration Research and Neuropathology Lab, Oslo, Norway; 30 LIED, University of Lübeck, Lübeck, Germany; 31 Department of Pharmacology, Medical Faculty, University of Latvia, Riga, Latvia; 32 Pathology Unit, Department of Molecular and Translational Medicine, University of Brescia Medical School, Brescia, Italy; 33 Medical Faculty, University of Belgrade, Serbia; 34 Department of Internal Medicine and Clinical Nutrition, Institute of Medicine at Sahlgrenska Academy, University of Gothenburg, Gothenburg, Sweden; 35 Department of Endocrinology, Sahlgrenska University Hospital, Gothenburg, Sweden; 36 Endocrinology, Abdominal Center, Helsinki University Hospital and University of Helsinki, Helsinki, Finland; 37 Department of Pathology, Rigshospitalet, Copenhagen University Hospital, Copenhagen, Denmark; 38 Department of Internal Medicine and Oncology, Faculty of Medicine, Semmelweis University, Budapest, Hungary; 39 Department of Pathological Cytology and Anatomy, Foch Hospital, Suresnes, France; 40 INSERM U1016, Institut Cochin, Paris, France; Université Paris Descartes-Université de Paris, Paris, France; 41 Department of Endocrinology, Sart Tilman B35, 4000 Liège, Belgium; 42 Department of Neurosurgery, Maria Sklodowska-Curie National Research Institute of Oncology, Warsaw, Poland; 43 Department of Endocrinology, Skåne University Hospital, Malmö, Lund University, Sweden

**Keywords:** ATRX (alpha thalassemia/mental retardation syndrome X-linked), aggressive PitNETs, pituitary carcinoma, pituitary adenoma, Cushing’s disease

## Abstract

**Context:**

Aggressive pituitary tumors (APTs) are characterized by unusually rapid growth and lack of response to standard treatment. About 1% to 2% develop metastases being classified as pituitary carcinomas (PCs). For unknown reasons, the corticotroph tumors are overrepresented among APTs and PCs. Mutations in the alpha thalassemia/mental retardation syndrome X-linked (*ATRX*) gene, regulating chromatin remodeling and telomere maintenance, have been implicated in the development of several cancer types, including neuroendocrine tumors.

**Objective:**

To study ATRX protein expression and mutational status of the *ATRX* gene in APTs and PCs.

**Design:**

We investigated ATRX protein expression by using immunohistochemistry in 30 APTs and 18 PCs, mostly of Pit-1 and T-Pit cell lineage. In tumors lacking ATRX immunolabeling, mutational status of the *ATRX* gene was explored.

**Results:**

Nine of the 48 tumors (19%) demonstrated lack of ATRX immunolabelling with a higher proportion in patients with PCs (5/18; 28%) than in those with APTs (4/30;13%). Lack of ATRX was most common in the corticotroph tumors, 7/22 (32%), versus tumors of the Pit-1 lineage, 2/24 (8%). Loss-of-function *ATRX* mutations were found in all 9 ATRX immunonegative cases: nonsense mutations (n = 4), frameshift deletions (n = 4), and large deletions affecting 22-28 of the 36 exons (n = 3). More than 1 *ATRX* gene defect was identified in 2 PCs.

**Conclusion:**

*ATRX* mutations occur in a subset of APTs and are more common in corticotroph tumors. The findings provide a rationale for performing ATRX immunohistochemistry to identify patients at risk of developing aggressive and potentially metastatic pituitary tumors.

Pituitary neuroendocrine tumors (PitNETs) (1), traditionally designated as pituitary adenomas, are usually benign tumors with indolent, nonaggressive course. Recently, the European Society of Endocrinology published criteria that define aggressive PitNETs as tumors demonstrating an unusually fast growth and/or lack of response to all standard treatment modalities including surgery, and radio- and pharmacological therapies (2). Pituitary carcinomas (PCs) are defined by the presence of noncontiguous craniospinal or distant metastases (3). While PCs are rare and constitute only 0.1% to 0.2% of all pituitary neoplasms (4), the prevalence of aggressive pituitary tumors (APTs) without metastases is less well known. An estimate of 3% has been suggested based on indices of increased proliferation and extensive p53 staining in tumor specimens from 451 patients reported to the German Pituitary Tumor Registry (5). Little is known about genetic abnormalities driving invasive and metastatic pituitary tumors. Whether they develop through malignant progression of benign pituitary tumors or occur as de novo malignant tumors caused by early, single, or multiple genetic changes predisposing for distant dissemination is unknown.

The functioning corticotroph tumors causing Cushing’s disease represent less than 5% of the benign, slow-growing PitNETs (6, 7). However, they are overrepresented among APTs and PCs, where they constitute approximately 30% to 40% (8, 9). One suggested explanation for this was a lower expression of the cell cycle inhibitor p27 in normal corticotroph cells and corticotroph tumors (10); however, the mechanisms are still unclear. Silent corticotroph tumors are also considered potentially more aggressive according to the current World Health Organization classification of the pituitary tumors (3), although a recent meta-analysis could not identify an increased recurrence rate in this subtype (11).

In patients with APTs, genetic abnormalities have previously only been reported in single sporadic cases, none has consistently been found in larger groups of patients (12). In a case of clinically nonfunctioning gonadotroph carcinoma, a low level of *HER2*/*neu* gene amplification was demonstrated by using fluorescence in situ hybridization and chromogenic in situ hybridization analysis (13). The presence of mi-RNAs probably targeting PTEN (phosphatase and tensin homolog) and TIMP2 (tissue inhibitor of metalloproteinases 2) was reported as potential drivers of metastatic growth in a case with a nonfunctioning PC (14). A single case of PC was reported in a patient with *succinate dehydrogenase subunit B* gene mutation and history of paraganglioma (15). Finally, tumor protein *p53* mutations in 2 PCs have been described (16).

Alpha thalassemia/mental retardation syndrome X-linked (ATRX) interacts with death domain-associated protein (DAXX) and the histone H3.3 variant in heterochromatin remodeling and maintenance of telomere structure and function (17, 18). Inactivation of ATRX or, less frequently, DAXX in* ATRX*/*DAXX* mutated tumors, leads to telomere destabilization and facilitates the process of alternative lengthening of telomeres (ALTs), which results in cancer cell immortality (19, 20). Somatic *ATRX* gene mutations are associated with several different tumor types, including astrocytomas in adults (21) and neuroendocrine tumors (NETs) such as pancreatic NETs (22, 23), neuroblastomas (24), and paragangliomas/pheochromocytomas (25, 26). Interestingly, in neuroendocrine tumors, *ATRX* abnormalities seem to predict malignant tumor phenotype, being present in high-grade malignant tumors such as neuroblastoma (24), or associated with poor prognosis and/or metastatic potential, such as in pancreatic NET (27), and pheochromocytomas/paraganglioma (26).

We have previously demonstrated normal immunohistochemical expression of ATRX protein in a large cohort of 246 well-characterized PitNETs localized to the sellar region, including 37 corticotroph tumors. However, 1 of 2 studied pituitary carcinomas (a corticotroph carcinoma in a patient with Cushing’s disease) did not express the protein due to a large deletion of the *ATRX* gene (28).

In the present study, we aimed to further explore ATRX protein expression and mutational status of the *ATRX* gene in a large cohort of aggressive PitNETs and pituitary carcinomas.

## Material and Methods

### Patient cohort

Pituitary tumor specimens were obtained from a multicenter cohort of 48 patients (15 female, 33 male), with a median age 45 (range 16-73 years) at diagnosis. Inclusion criteria were at least 1 pituitary surgery and tumor progression despite radiotherapy, and/or while on treatment with dopamine agonists or somatostatin analogues, or metastatic disease. Thirty patients had APTs and 18 had PCs with cerebrospinal and/or systemic metastases. The median time from diagnosis of the pituitary tumor to metastases was 8.5 (range 1.2-36) years (Table 1). The patients were treated at specialized centers in 11 European countries (Belgium, Denmark, Finland, France, Hungary, Italy, Norway, Poland, Serbia, Sweden, and UK). Patients’ data and tumor characteristics at the first presentation, treatments given, and outcome were collected in anonymized standardized questionnaires filled in by all participating centers.

**Table 1.** Patient and tumor characteristics in the study population

	Total	APT	PC
Total n	48	30	18
Age at diagnosis, year (median, range)	45 (16-73)	46.5 (18-73)	42 (16-69)
Male n (%)	33 (69)	23 (77)	10 (56)
Macroadenomas^*a*^	44/45	28/29	16/16
Invasive growth^*a*^	39/42	24/27	15/15
No of surgeries (median, range)	3 (1-10)	3 (1-10)	3.5 (1-8)
No of radiotherapies (median, range)	1 (0-4)	1 (0-2)	2 (1-4)
Resistance to DA/ somatostatin analogs^*b*^	27/27	18/18	10/10
Time to metastases from first surgery, year (median, range)			8.5 (1.2-36)
Treatment with cytotoxic drugs^*b*^	35/37	21/23	14/14
ATRX negative, n (%)	9 (19)	4 (13)	5 (28)
Tumor subtypes (IHC)			
Corticotroph^*c*^	22	10	12
Lactotroph	15	12	3
Somatotroph	4	2	2
Somato/lactotroph	2	1	1
TSH/FSH	1	1	0
Silent Pit 1 positive PitNET	3	3	0
Null cell	1	1	0

Abbreviations: APT, aggressive pituitary tumor; PC, pituitary carcinoma; DA, dopamine agonist; IHC, immunohistochemistry.

^*a*^MRI at first tumor presentation in patients with available information.

^*b*^In patents with available information.

^*c*^Six clinically silent (2 PCs, 4 APTs).

Information on pituitary tumor size and local extension at the first magnetic resonance imaging (MRI) was available in 45 and 43 patients, respectively. All but 1 lactotroph tumor were macroadenomas at the time of diagnosis. By the time of pituitary surgery, invasion of the cavernous sinuses, bone and/or brain was evident on MRI in the 39 cases, including the single patient who had a microadenoma. Of the 48 patients, 39 had more than 1 pituitary surgery, and 33 more than 2. Forty-six out of the 48 patients had received at least 1 radiotherapy. In 1 case, tumor size and extension were considered too large for radiotherapy, and in the second case the reason for not performing radiotherapy was not available. No tumor treated with dopamine agonists and/or somatostatin analogues (octreotide, lanreotide, pasireotide) was controlled by these medications (Table 1). In addition to standardized medical therapy, 34 patients had received treatment with chemotherapy, temozolomide in 33 including 1 patient with additional bevacizumab, and another 1 with an mTOR inhibitor and 2 immune checkpoint inhibitors.

Tumors were classified based on the laboratory and clinical signs of pituitary hormone hypersecretion, expression of anterior pituitary hormones in the tumor cells, and, in the cases of hormone-negative nonfunctioning tumors, by their expression of pituitary-specific transcription factors. Corticotroph tumors were the most common: 22/48, of which 16 were functioning tumors causing Cushing’s disease. Lactotroph tumors were the second most common, n = 15 (Table 1).

The index patient with *ATRX* mutation has been previously reported (28) and is also included in the present study. Of the 48 patients, 3 had syndromes predisposing for pituitary tumors, 1 had MEN1 (29), 1 had Lynch syndrome (30), and 1 patient belonged to a kindred with familial predisposition for pituitary tumors, but without *MEN1* or *AIP* mutation. In addition, pituitary tumor tissue from a corticotroph nonaggressive macroadenoma in a patient with Lynch syndrome was investigated. This case was not included in the statistical analyses as it did not fulfil the criteria for aggressive tumors.

In 45 patients, at least 1 specimen from pituitary surgery was available for analyses. In the remaining 3 patients, there was only specimen from the metastasis. For 7 patients with carcinoma, material from both pituitary surgery and from metastatic tumor was available. The presence of representative tumor tissue was confirmed in hematoxylin and eosin stained slides from all specimens.

### Immunohistochemical analyses

Immunohistochemistry (IHC), with antibodies towards growth hormone (GH), prolactin (PRL), thyrotroph hormone (TSH), adrenocorticotroph hormone (ACTH), gonadotroph hormones, follicle-stimulating hormone (FSH), and luteinizing hormone (LH), was performed at the local IHC laboratories according to the routine protocols. Immunohistochemical analysis with antibodies towards pituitary-specific transcription factors was performed at Uppsala University Hospital by using anti-SF1 antibody (Abcam, ab217317), anti-Pit-1 antibody (Novus Biologicals, NBP1-92273), and anti-T-Pit antibody (Atlas Antibodies, AMAb91409), according to the standard protocols.

ATRX protein expression was studied on whole sections from formalin-fixed paraffin-embedded tissue blocks. For the patients operated on more than once, available tissue specimens from multiple surgeries were examined. In the majority of cases, IHC was performed at Uppsala University Hospital in a DAKO-Autostainer Link 48 with heat-induced epitope retrieval at high pH. Purified polyclonal anti-ATRX antibody (HPA001906, Atlas Antibodies; dilution 1:100; incubation time 20 minutes) was used. Specimens from 2 adult astrocytomas, 1 with *ATRX* mutation and 1 without *ATRX* mutation, both confirmed by using molecular genetic analysis, were used as negative and positive controls. In addition, immunolabelled endothelial cells served as an internal positive control. Four cases from Foch Hospital (Suresnes, France) and a case from University Hospital in Copenhagen, Denmark, were stained in Ventana Benchmark by using the same antibody and according to the locally optimized protocols.

### Molecular genetic analysis

Molecular genetic analysis was performed on tumor tissue from the pituitary specimen in all nine cases demonstrating lack of ATRX immunolabelling. In 2 patients, specimens from metastases were also analyzed. If there was more than 1 specimen from the pituitary surgery, the specimen with the most representative tumor tissue was used. In 1 patient, a partial lack of ATRX protein labelling was observed in the pituitary specimen and a total lack in metastatic tumor tissue. In this patient, an attempt was made to microdissect tissue and extract DNA separately from ATRX negative and positive area of the pituitary tumor. In addition, the specimen from metastasis with negative ATRX staining was analyzed. All but 1 specimen were examined by a next-generation sequencing (NGS) panel targeting 20 genes (31) related to cancers of the central nervous system as in the initial study (28). The proportion of tumor cells exceeded 70% in all the specimens. One specimen was analyzed using an exome-wide sequencing approach.

### Next-generation sequencing

DNA was purified from 10-µm paraffin slides using GeneRead DNA FFPE Kit (Qiagen, Germany) according to the manufacturer’s instructions. NGS was performed with a custom designed central nervous system panel covering the entire coding sequence or hotspot regions of 20 genes frequently mutated in brain tumors (32). DNA was quantified using an RNase P TaqMan Copy Number Reference Assay performed on a QuantStudio 12K Flex Real-Time PCR System (Applied Biosystems, Foster City, CA). Libraries were prepared in 2 primer pools using the Ion AmpliSeq Library Kit Plus and Ion Xpress Barcode Adapters 1–96 Kit in 10 µL of reaction volume with 5 ng of template DNA. Library quantitation was performed using the Ion Library Quantitation Kit. Sample preparation, chip loading, and sequencing were performed using Ion Chef and Ion Torrent S5 System with Ion S5 Chef solutions, Ion S5 sequencing reagents and Ion 530/540 Chip Kits. All Ion products were supplied by Ion Torrent/ThermoFisher Scientific, Carlsbad, CA, USA. Data analysis, including base calling, quality scoring, trimming, demultiplexing, and alignment, was performed using standard Ion Torrent Suite v5.10 workflows. BAM alignment files were manually analyzed for alterations in the coding sequences of the 20 genes using Golden Helix GenomeBrowse 3.0 (Golden Helix, Bozeman, MT, USA). The sequencing experiments included *ATRX* wild-type control samples from healthy donors.

One specimen was analyzed using hybridization capture-based high-throughput NGS platform from Illumina (33).

### Ethics approval

The study has been approved by Regional Ethics Committee in Uppsala (Dnr 2018/327).

## Results

### Lack of ATRX protein expression is frequent in corticotroph tumors

Nine of the 48 tumors (19%) demonstrated lack of ATRX immunolabelling in the tumor cells. Five were carcinomas and 7 were corticotroph tumors, representing 32% of all corticotroph tumors (7 out of 22). Lack of protein expression was more common in patients with functioning corticotroph tumors (6/16, 38%) than in those with silent corticotroph tumors (1/6, 17%). Of the remaining 2 ATRX-immunonegative tumors, 1 was a lactotroph APT with a fatal outcome, and 1 was a somato-lactotroph carcinoma that initially presented as a prolactinoma and subsequently evolved into acromegaly (Table 2).

**Table 2.** Patient and tumor characteristics in ATRX mutated vs intact cases

	ATRX mutated	ATRX intact
Total n	9	39
Age at diagnosis, year (median, range)	45 (23-72)	45 (16-73)
Male, n (%)	6 (67)	27 (69)
Aggressive pituitary tumors, n (%)	4 (44)	26 (67)
Pituitary carcinomas, n (%)	5 (56)	13 (33)
Tumor subtypes (IHC)		
Corticotroph (n = 22)	7	15
PC (n = 12)	4	8
APT (n = 10)	3	7
Lactotroph (n = 15)	1	14
PC (n = 3)	0	3
APT (n = 12)	1	11
Somato/lactotroph (n = 2)	1	1
PC (n = 1)	1	0
APT (n = 1)	0	1
Other subtypes^*a*^ (n = 9)	0	9
PC (n = 2)	0	2
APT (n = 7)	0	7

Abbreviations: APT, aggressive pituitary tumor, IHC, immunohistochemistry; PC, pituitary carcinoma.

^*a*^Somatotrophs (4); silent Pit 1 positive (3); double TSH/FSH (1); null cell PitNET (1).

More than 1 pituitary specimen was available for analysis in 6 of 7 patients who underwent multiple surgeries. In 5 of the 6 patients, all specimens demonstrated lack of ATRX in all tumor cells. In 1 patient, the specimen from the first surgery could not be assessed, and there was partial lack of ATRX expression in pituitary tumor from the second surgery and a total lack in the metastasis. In 5 patients with PC, specimens from metastases were available in 4 and demonstrated negative ATRX staining in the tumor cells. The remaining 39 pituitary tumors demonstrated intact nuclear ATRX expression.

**Figure 1.**
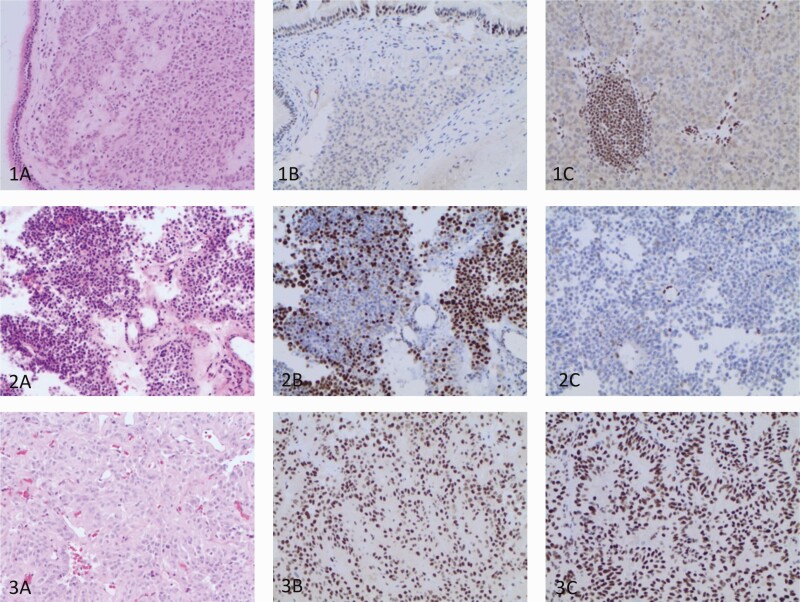
Histopathological and immunohistochemical features of PitNETs. Row 1: Hematoxylin eosin staining of a primary pituitary tumor invading into the respiratory mucosa (1A) with a total lack of ATRX in tumor cells nuclei both the primary pituitary tumor (1B) and a lymph gland metastasis (1C) in a patient with a functioning somato-lactotroph carcinoma. ATRX expression is intact in respiratory epithelium, endothelial cells and lymphocytes. Row 2: Hematoxylin eosin staining from the primary pituitary tumor in a patient with a silent corticotroph carcinoma (2A). A partial nuclear ATRX-loss in a proportion of cells in the specimen from the second pituitary surgery (2B) and a total ATRX-loss in the metastasis (2C) ATRX expression is preserved in the nuclei of the endothelial cells. Row 3: Hematoxylin eosin staining of the specimen from the first surgery (3A) and normal ATRX expression in the nuclei of the tumor cells in the specimens from 2 pituitary surgeries (3B, 3C) in a patient with a silent Pit-1 positive PitNET.

Examples of PitNETs with normal ATRX staining, total lack of immunolabelling and partial negative ATRX staining in primary and metastatic tumors are illustrated in Fig. 1.

### All ATRX-immunonegative tumors harbor loss-of-function *ATRX* gene abnormalities

**Figure 2.**
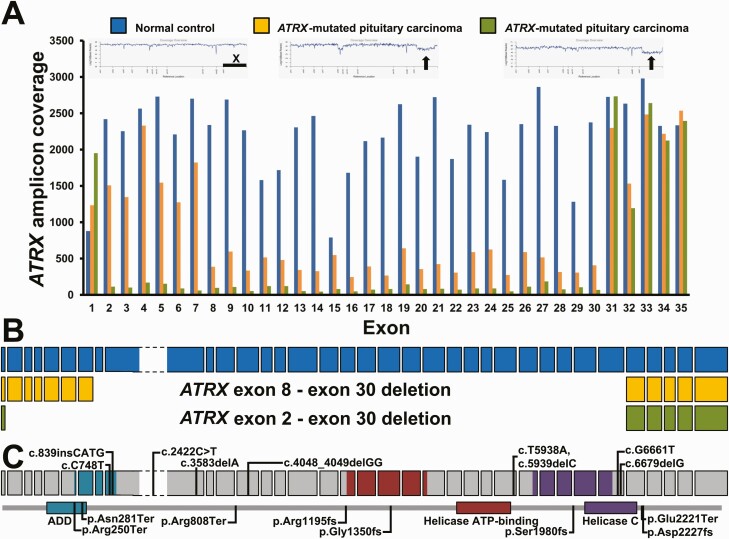
ATRX deletions in 2 patients with corticotroph carcinoma. (A) Profile of the average amplicon coverage of ATRX coding sequence from exon 1 through exon 35. A healthy donor of female origin was included in all NGS experiments (blue). Low amplicon coverage corresponding to deletion of ATRX sequence was observed for 2 patients, highlighted with orange and green, respectively. Coverage overviews are shown as inserts. The black horizontal bar indicates the position of chromosome X. Arrows mark the deletions in ATRX. (B) Schematic illustration of the 2 large, intragenic ATRX deletions spanning exon 2/exon 8 through exon 30. The large exon 9 of ATRX, depicted with stippled lines, is compressed for clarity. (C) Diagram of ATRX variants at coding and protein levels, respectively. Definition of ATRX domains were from UniProt. ADD, ATRX-DNMT3-DNMT3L; Helicase C, Helicase C-terminal.

*ATRX* loss-of-function gene abnormalities were found in all 9 ATRX-immunonegative tumors (Table 3) (31). Two different damaging *ATRX* mutations with large differences in mutation frequencies were identified in the same primary tumor in 2 carcinomas from male patients. One of these 2 tumors demonstrated a partial lack of ATRX at IHC. An attempt to extract separately DNA from ATRX-immunopositive and negative fraction was, however, unsuccessful, as the same mutational status was confirmed in both fractions. Interestingly, only the predominant mutation from this pituitary tumor was present in the metastasis (6 years later) with a frequency of 98%, suggesting clonal heterogeneity and evolution of the primary tumor (Table 3) (31). Three tumors did not show any *ATRX* single nucleotide variants or small indels, but had large, intragenic deletions corresponding to most of the coding sequences (22-28 of 36 exons) (Fig. 2A and 2B). One of these tumors was the corticotroph tumor previously reported, whereas the other 2 were lactotroph and somato-lactotroph, respectively. All identified *ATRX* single nucleotide variants and small indels were positioned throughout the coding sequence of the *ATRX* gene (Fig. 2C). In addition to the *ATRX* mutations, 8 out of 9 ATRX-immunonegative tumors had other genetic abnormalities: inactivating somatic mutations in tumor suppressor genes *TP53* (6), *PTEN* (2), *RB1* (1), *NF2* (1), and a homozygous deletion of *CDKN2A/B* in both primary tumor and metastasis in 1 patient (Table 3). Recurrent copy number variants (CNVs) that were estimated from the sequencing data were all gains, and involved chromosomes 5, 7, 9p21.3 encompassing *CDKN2A/B* loci as well as the CIC locus on 19q.

**Table 3.** Genetic alterations in ATRX-negative APT and PC by panel NGS

Pt.	Specimen	Local.	ATRX expression	Genes	Coding	Amino Acid	Freq. (%)#
1	Cushing/PC	Pituitary	loss	*ATRX*	c.134_6217del	p.D45-K2027del	Nu
2	Cushing/PC	Pituitary	loss	*ATRX*	c.748C>T	p.Arg250Ter	89
	Lynch sy			*TP53*	c.524G>A	p.Arg175His	84
				*PTEN*	c.697C>T	p.Arg233Ter	10
3	Lactotroph/APT	Pituitary	loss	*ATRX*	c.21_6699del	p.E8-K2233del	Nu
				*TP53*	c.584T>A	p.Ile195Asn	92
				*RB1*	c.1725_1726 insAACAA	p.Ser576fs	13
				*RB1*	c.1218_1697del	p.N406-S565del	He
4	Cushing/PC	Pituitary	loss	*ATRX*	c.6679delG	p.Asp2227fs	81
				*ATRX*	c.3583delA	p.Arg1195fs	12
5*	Silent ACTH/PC	Pituitary	retained (major)/loss (minor)	*ATRX*	c.4048_4049delGG	p.Gly1350fs	28
				*ATRX*	c.6661G>T	p.Glu2221Ter	31
				*TP53*	c.644G>A	p.Ser215Asn	30
5^*a*^	Silent ACTH/PC	Pituitary	loss (major)/retained (minor)	*ATRX*	c.4048_4049delGG	p.Gly1350fs	67
				*ATRX*	c.6661G>T	p.Glu2221Ter	10
				*TP53*	c.644G>A	p.Ser215Asn	8
5	Silent ACTH/PC	Metastasis	loss	*ATRX*	c.4048_4049delGG	p.Gly1350fs	98
6	Cushing/APT	Pituitary	loss	*ATRX*	c.2422C>T	p.Arg808Ter	72
				*TP53*	c.1024C>T	p.Arg342Ter	51
				*PTEN*	c.697C>T	p.Arg233Ter	55
7	Cushing/APT	Pituitary	loss	*ATRX*	c.839_840insCATG	p.Asn281Ter	44
				*TP53*	c.818G>A	p.Arg273His	85
				*NF2*	c.1052G>A	p.Arg351His	20
8	Cushing/APT	Pituitary	loss	*ATRX*	c.5938T>A, c.5939delC	p.Ser1980fs	88
				*TP53*	c.375G>A	p.( = )	81
9	Mixed GH-PRL/PC	Pituitary	loss	*ATRX*	c.595_6699del	p.N199-K2233del	He
				*CDKN2A*	c.1_501del	p.M1-A167del	Ho
				*CDKN2B*	c.1_414del	p.M1-D138del	Ho
9	Mixed GH-PRL/PC	Metastasis	loss	*ATRX*	c.595_6699del	p.N199-K2233del	He
				*CDKN2A*	c.1_501del	p.M1-A167del	Ho
				*CDKN2B*	c.1_414del	p.M1-D138del	Ho

Abbreviations: APT, aggressive pituitary tumors; PC, pituitary carcinoma; NGS, next-generation sequencing; ACTH, adrenocorticotropic hormone, GH, growth hormone; PRL, prolactin; #, estimated ploidy level of larger gene deletions: Nu: nullizygous, He: hemizygous deletion, Ho: homozygous deletion.

^*a*^The same mutations were detected in ATRX immunopositive and immunonegative tissue fractions indicating that they could not be successfully separated.

## Discussion

Little is known about genetic abnormalities driving invasive and metastatic growth of PitNETs. Here, we demonstrate a loss of ATRX protein expression caused by severe loss-of-function *ATRX* gene alterations in almost a fifth of highly APTs, with a higher prevalence in PC than in APT, and in corticotroph tumors than in other lineage subtypes. This indicates that corticotroph tumors are prone to develop *ATRX* gene abnormalities.

We reported previously normal ATRX expression in 246 PitNETs localized to the sellar region. However, in 1 female patient diagnosed with Cushing’s disease and a pituitary macroadenoma at an age of 36 years, we found negative ATRX immunolabelling caused by a large deletion of the *ATRX* gene (28). This tumor had progressed over time and had become metastatic despite multiple transsphenoidal surgeries, pharmacological therapy, and 3 different modalities of radiation therapy. ATRX staining was absent in all the tumor specimens including the 1 from the first surgery.

In the present extended study, we demonstrate *ATRX* gene defects in 8 additional patients. Thus, 9 out of 48 patients (19%) with APTs or carcinomas harbored loss-of-function *ATRX* gene alterations, more frequently in patients with PC than with APT (28% vs 13%). Five out of the total 9 patients with *ATRX* gene defects had carcinomas. Of the 4 APT patients, 2 died due to progressive tumor growth, in another there was a short time from the tumor diagnosis to the study end, and in the last patient search of metastases was not performed due to advanced dementia. Further studies with longer follow-up are needed to assess to what extent an initial *ATRX* defect leads to a metastatic disease.

In addition to our previously reported case of *ATRX* mutated corticotroph carcinoma (28), a corticotroph carcinoma with an *ATRX* mutation in combination with *PTEN* and *TP53* mutations has been described; however, without detailed presentation of genetic data (34).

In a recent study (35), whole exome sequencing of 18 corticotroph tumors lacking mutations in the *USP8* (ubiquitine specific peptidase 8) gene, mutations that drive corticotroph tumors in approximately 50% of patients with Cushing’s disease, demonstrated *ATRX* mutations concomitantly with *TP53* mutations in 2. Although detailed clinical data regarding aggressiveness of the 2 *ATRX* mutated tumors were not presented, both were recurrent and required surgery on 2 and >3 occasions, respectively, and Ki67 proliferative index was increased in 1 of the cases (35). Lack of ATRX immunolabelling was recently found in 3 lactotroph macroadenomas from a cohort of 42 pediatric PitNETs, but molecular genetic confirmation of the *ATRX* mutations was not provided (36). Recently, ALT phenotype has been reported in 3 of 106 PitNETs, 2 were recurrent nonfunctioning PitNETs without specification of cell linage differentiation, and 1 was a somatotroph tumor (37). Two of the 3 ALT-positive PitNETs demonstrated loss of ATRX or DAXX at protein level, indicating a homozygous loss of the gene or alternative mechanism of gene silencing. However, no *ATRX* or *DAXX* mutations were identified by sequencing (37).

In patients who had repeated pituitary surgeries in the present cohort, an *ATRX* defect was already present in the first removed tumor, though in 1 patient tumor tissue from the first surgery was not evaluable. This indicates that *ATRX* abnormalities represent an early genetic event contributing to aggressive behavior and, at least in a subset of patients, to metastatic spread. Where material from both the pituitary tumor and metastasis was available (n = 4), identical patterns of a complete loss of ATRX were seen in 3, whereas 1 one case, partial loss of ATRX was identified in the pituitary tumor and a complete loss in the metastasis. A similar case of a PitNET with ALT-negative phenotype in the original tumor, and ALT-positive phenotype and a partial loss of ATRX in a recurrent tumor, was recently reported (37). These findings suggest that an *ATRX* mutation may occur, though rarely, in pituitary tumors with primarily intact *ATRX*, contributing to malignant tumor progression.

In the *ATRX*-mutated cases in our cohort, we demonstrated different loss-of-function *ATRX* defects including nonsense mutations, frameshift indels, and, in 3 cases, large, intragenic deletions of almost the whole gene (22-28 of the 36 exons). Interestingly, large deletions of almost the whole *ATRX* gene have only rarely been reported in other tumor types, such as astrocytomas (21, 32), pancreatic NETs (22), and pheochromocytomas and paragangliomas (25). Yet, a recent study on *ATRX* alterations in neuroblastoma demonstrated a strong tendency for large, intragenic deletions of exons 1-9, encoding the first half portion of the ATRX protein (38). In our cohort, there was no predominance of a particular type of mutation in carcinomas compared with APTs, or in corticotroph compared with Pit-1-lineage tumors. However, the number of mutated cases may be too low to make conclusions on a potential genotype–phenotype association.

Blood samples or normal tissues from patients were not included in the sequencing experiments to test for germline mutations. The variant allele frequencies of mutations in *ATRX* reported in this study are in favor of somatic rather than germline origin. Furthermore, IHC revealed normal ATRX expression in non-neoplastic cells in all the mutated specimens, arguing for the somatic origin of the *ATRX* gene defects.

In the present study, we had the opportunity to investigate ATRX in 2 patients with corticotroph tumors, 1 nonaggressive macroadenoma and 1 carcinoma, and Lynch syndrome, a cancer predisposing syndrome with mutations in genes involved in DNA mismatch repair (*MLH1*, *MSH2*, *MSH6*, *PMS2*, *EPCAM*). Both tumors harbored an *MSH2* mutation, but only the severe case, a carcinoma, in addition exhibited an *ATRX* mutation.

Additional cancer-related mutations were identified and associated with *ATRX* alterations in 8 of 9 cases, *TP53* mutations in 6 (3 aggressive corticotroph tumor, 2 corticotroph carcinomas, and 1 aggressive lactotroph tumor), *PTEN* mutations in 2, and *RB1*, *NF2*, and *CDKN2A/B* in single cases. *TP53* mutations have rarely been previously reported in pituitary tumors (16). However, recently, *TP53* mutations were demonstrated in 6 out of 18 of corticotroph *USP8* wild-type tumors and correlated with larger tumors and higher Ki67 index (35). Our findings, together with previous report, may suggest an association of the *TP53* mutations with corticotroph tumors with more aggressive phenotype. Findings of multiple mutations in the *ATRX* mutated tumors may indicate genetic instability leading to multiple cancer-related genetic events. However, more extensive molecular genetic analyses are needed to get full insight into genetic landscape of aggressive PitNETs.

The strength of the present study is the well characterized cohort of APT and PC and a relatively large number of patients, having in mind the rarity of the condition. A limitation is a short follow-up of some of the patients with *ATRX* defects, which limits conclusions on the metastatic potential of this mutation.

Although many APT/carcinomas exhibit histological features consistent with increased proliferation (Ki-67 index > 3%, increased mitotic count, and p53 expression) (4), and coexistence of 2 of the 3 markers is associated with increased risk of tumor progression and recurrence (39), the presence of these features does not fully predict future aggressive behavior (40, 41). To our knowledge, the present findings is the first time that a gene mutation with well-known oncogenic potential has been consistently reported in a proportion of aggressive PitNETs.

Currently, temozolomide is the first-line chemotherapy for APT and PC (29). The drug induces an initial response rate of 40%, but subsequently most tumors relapse and long-term effective alternative therapies are still lacking (42). Mutated *ATRX* is an attractive therapeutic target for the subgroup of ATRX negative pituitary tumors. There is ongoing intensive research aiming to develop pharmacological therapies targeting *ATRX* and ALT (43, 44).

In summary, the results of this study provide a rationale for performing ATRX immunohistochemistry as a simple, inexpensive, and widely available laboratory test to identify patients at increased risk for development of highly aggressive and potentially metastatic PitNETs, especially in macroadenomas causing Cushing’s disease or in clinically silent corticotroph tumors. Patients with pituitary tumors harboring an *ATRX* mutation should be offered closer follow-up, including work-up for metastatic dissemination, and invasive treatment at the early stages of the disease.

## Data Availability

Some or all data generated or analyzed during this study are included in this published article or in the data repositories listed in References.

## Abbreviations

ACTH: adrenocorticotroph hormone
ALT: alternative lengthening of telomere
APT: aggressive pituitary tumor
ATRX: alpha thalassemia/mental retardation syndrome X-linked
DAXX: death domain-associated protein
FSH: follicle-stimulating hormone
GH: growth hormone
IHC: immunohistochemistry
LH: luteinizing hormone
MRI: magnetic resonance imaging
NET: neuroendocrine tumor
NGS: next-generation sequencing
PC: pituitary carcinoma
PitNET: pituitary neuroendocrine tumors
PRL: prolactin
PTEN, PTEN: phosphatase and tensin homolog
TSH: thyrotroph hormone

## Additional Information

***Disclosure Summary*:** The authors have nothing to disclose.
